# On the estimation of brain signal entropy from sparse neuroimaging data

**DOI:** 10.1038/srep23073

**Published:** 2016-03-29

**Authors:** Thomas H. Grandy, Douglas D. Garrett, Florian Schmiedek, Markus Werkle-Bergner

**Affiliations:** 1 Center for Lifespan Psychology, Max Planck Institute for Human Development, Berlin, Germany; 2 Department of Neurology, Charité—University Medicine, Berlin, Germany; 3 Max Planck-UCL Centre for Computational Psychiatry and Ageing Research, Max Planck-University College London Centre for Computational Psychiatry and Ageing Research, Max Planck Institute for Human Development, Lentzeallee 94, 14195 Berlin, Germany; 4 German Institute for International Educational Research, Frankfurt am Main, Germany

## Abstract

Multi-scale entropy (MSE) has been recently established as a promising tool for the analysis of the moment-to-moment variability of neural signals. Appealingly, MSE provides a measure of the predictability of neural operations across the multiple time scales on which the brain operates. An important limitation in the application of the MSE to some classes of neural signals is MSE’s apparent reliance on long time series. However, this sparse-data limitation in MSE computation could potentially be overcome via MSE estimation across shorter time series that are not necessarily acquired continuously (e.g., in fMRI block-designs). In the present study, using simulated, EEG, and fMRI data, we examined the dependence of the accuracy and precision of MSE estimates on the number of data points per segment and the total number of data segments. As hypothesized, MSE estimation across discontinuous segments was comparably accurate and precise, despite segment length. A key advance of our approach is that it allows the calculation of MSE scales not previously accessible from the native segment lengths. Consequently, our results may permit a far broader range of applications of MSE when gauging moment-to-moment dynamics in sparse and/or discontinuous neurophysiological data typical of many modern cognitive neuroscience study designs.

The human brain is remarkably dynamic and variable across moments, no matter how brain signals are measured (e.g., ECoG, EEG, MEG, fMRI). The utility of considering signal variability to understand individual differences (e.g., in cognition, age, disease) continues to gain traction in the neuroimaging community, to the point that signal variability measures often outpredict traditional (e.g., mean signal-based) measures in a host of experimental contexts^1^. Importantly, signal variability and dynamics may serve as proxies for healthy, efficient, flexible neural systems^1^,^2^. However, *in vivo* studies remain relatively sparse, with much future work required to better characterize the form and function of signal variability in the human brain. Although various signal variability metrics have been utilized, multi-scale entropy^3^,^4^ (MSE)—an extension of sample entropy^5^ (SampEn) that estimates SampEn at multiple time scales—is one popular measure recently applied as a tool for quantifying the (nonlinear) predictability of neural signals^6^,^7^,^8^,^9^,^10^,^11^,^12^. SampEn indexes the presence of patterns in the data; signals with a repetitive structure (like a sine wave) have lower entropy, and less predictable (or random) signals have higher entropy. Importantly, the broader utility of the multi-scale extension of SampEn (i.e., MSE) lies in its ability to account for entropy within different time scales (fast to slow) on which the brain operates, similar in logic to the use of multiple frequencies in the Fourier or Wavelet domains^13^,^14^.

However, one of the primary challenges in using MSE to characterize variability in human brain data is the apparent need for substantial continuous data for robust estimation^4^. Heuristically, the recommended number of successive data points for estimation at *each* scale is 100 (minimum) to 900 (preferred) points^15^ using typical MSE parameter settings, although several neuroimaging papers also have used as little as 50 points as the minimum^7^,^8^,^9^,^10^. Nevertheless, this data density requirement has so far precluded applicability of the MSE measure to experimental designs in which continuous data are too few for stable MSE estimation. For example, in a standard fMRI block design with a TR of 2 sec and block lengths of 0.5–1 min, the number of continuous data points (15–30 data points) is purportedly too sparse for MSE estimation from even a single temporal scale. In a similar vein, in an epoch-based ECoG/EEG/MEG study, characterization of the lower frequency range may not be achievable. For instance, for an epoch length of 1 sec, following the recommendation that MSE be estimated from at least 100 continuous data points restricts the scales to “sampling frequencies” of at least 100 Hz (i.e. 100 data points/sec). Thus, for segments as short as 1 sec, estimating the MSE at scales fully capturing the alpha^16^,^17^,^18^ (8–12 Hz) and theta^19^,^20^ (4–7 Hz) frequency range is severely limited. Consequently, MSE data density requirements seemingly preclude most standard non-resting-state fMRI studies (event- or block-designs), and have limited applicability in epoch-based ECoG/EEG/MEG studies. In short, for MSE to usefully characterize signal dynamics within typical cognitive neuroscience study designs, alternative solutions are required to relax high data density requirements.

In the current paper, we investigated whether this potential limitation of MSE can be overcome by estimation of MSE across *discontinuous* segments of a time series (see Fig. 1). From a theoretical point of view, estimation of MSE across discontinuous segments of neural signals should not pose a fundamental problem, under the assumption of neurophysiological equivalence of data segments (i.e., that data segments are all equivalent samples of a signal with specific signal characteristics). MSE—like any classic entropy measure—is intrinsically discrete in that it compares the relative occurrence and predictability of patterns of neighboring data points. In principle, this pattern counting process should be extensible to discontinuous data segments, so long as data patterns are not computed across segment boundaries (doing so would result in artificial “patterns” that are not meaningful to and representative for the signal characteristic under investigation). Thus, we hypothesized that by taking a continuous time series and parsing it into various data segments (Fig. 1A), MSE values calculated across the discontinuous time segments would mirror MSE values from the continuous time series. Further, by leveraging multiple continuous data segments, it should also be possible to estimate MSE scales that would otherwise not be attainable due to a lack of continuous within-segment data (so long as there is enough total data across segments to stabilize estimates, and patterns are not counted across boundaries). If so, the applicability of MSE would extend to study designs that contain only segments of limited continuous data. We provide support for this hypothesis using simulated, EEG, and fMRI data.

## Methods

### Multiscale entropy (MSE)

MSE is a measure of the predictability of a signal at multiple time scales^3^,^4^. Typically, MSE relies on the estimation of the sample entropy^5^ (SampEn) at each time scale of interest. In simple terms, the estimation of SampEn within a given time series involves counting how often patterns of *m* successive data points reoccur in time (*U*^*m*^), and counting how often patterns of *m* + 1 data points reoccur in time (*U*^*m*+1^). Importantly, given that data points are rarely equal in physiological time series, a criterion is required to define when a data pattern has “reoccurred.” A parameter *r* is set that represents a proportion of the time series standard deviation (*SD*), thus defining a boundary within which data points are considered “equal”; this in effect discretizes the data, allowing for comparison of “data patterns” rather than exact data values. That is, for any data point *k*, all data points within *k* ± *r* × *SD* are by definition equal to *k*. SampEn is then given as the natural log of *U*^*m*^(*r*)/*U*^*m*+1^(*r*). Consequently, high SampEn values indicate low predictability (or higher randomness) of data patterns by indicating that patterns of length *m* + 1reoccur less often than patterns of length *m* (i.e., the higher the ratio *U*^*m*^(*r*)/*U*^*m*+1^(*r*), the higher SampEn and vice versa). The inclusion of multiple time scales in MSE^3^,^4^ is achieved by coarse-graining the original time series prior to SampEn estimation: for any scale *s*, *s* successive, non-overlapping data points are averaged, and SampEn is calculated across these averaged data points. As MSE at scale 1 is identical to SampEn, we will refer to SampEn whenever MSE analyses are focused on scale 1. The boundary criterion *r* × *SD* is typically defined only once for the native time series (i.e., scale 1) and then applied to all MSE scales. Accordingly, MSE may be interpreted as the SampEn at each scale relative to the overall variance (*SD*) of the time series. Importantly, each MSE scale must necessarily be interpreted in the context of the sampling rate. That is, scale 1 represents the native sampling rate of a given digitized signal (e.g., 250 Hz, or 4 ms sampling interval), scale 2 represents the native sampling rate/2 (e.g,. 125 Hz, or 8 ms), and so on for successive scales. Thus, scale-specific MSE results can only be compared across studies when accounting for differences in sampling rate.

Typically, in neurophysiological settings, parameter values of *m* = 2 and *r* = 0.50 are utilized^6^,^8^,^9^. Although *m* (also referred to as the embedding dimension) is often set to 2, optimal values can also be determined using the procedure proposed by Small and Tse^21^. *r* value settings of 0.50 are also typically specified according to recommendations by Richman and Moorman^5^. In the current paper, we mainly report analyses using these specific parameters (analyses with *r* parameters of 0.15 and 0.30 did not reveal any substantial differences in our analyses; see Figures S1 and S2). Calculation of MSE was implemented as described in Costa *et al.*^4^. For the calculation of the MSE across discontinuous segments, two further constraints were added: (1) coarse-graining was conducted segment-wise (i.e., within segments), and (2) patterns were not allowed to cross segment borders. Thus, in addition to the requirements on the number of data points as described above, the maximal scale possible was restricted here to be [*n*/(*m* + 1)] rounded down to the nearest integer, with *n* being the number of successive data points per segment.

### Evaluation of SampEn and MSE estimates from sparse data

**Figure 1:**
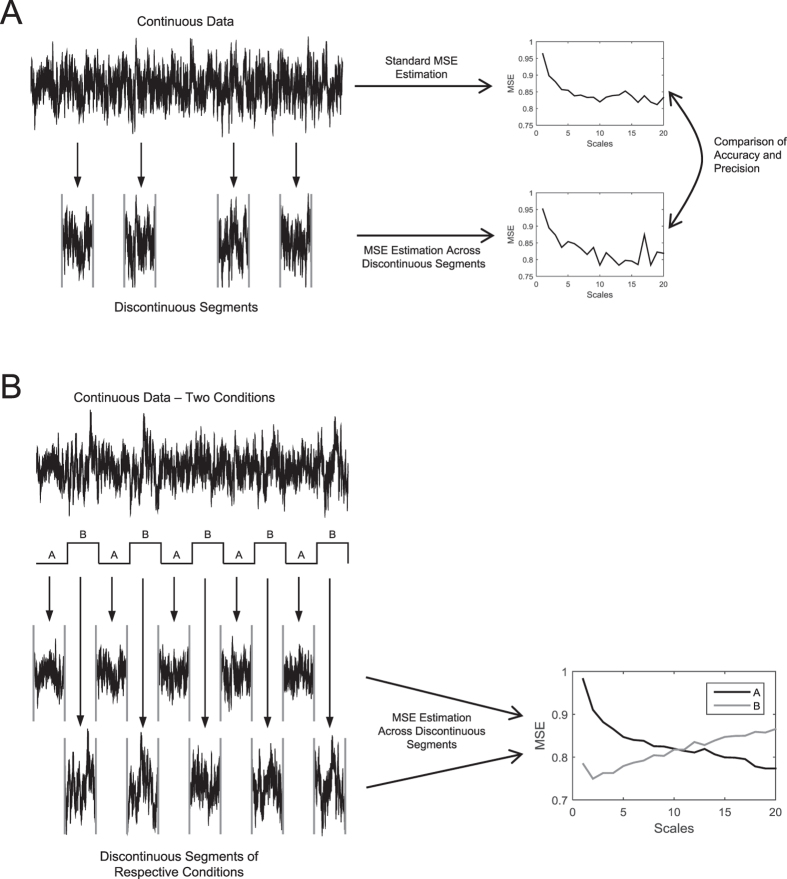
Schematic representation of the rationale of MSE estimation across discontinuous segments of a time series. (**A**) Sampling of discontinuous segments from a continuous time series and subsequent comparison of MSE estimates across discontinuous segments with MSE estimates from continuous time series. Here, *accuracy* denotes the absolute deviation of (a) MSE values from discontinuous segments from (b) the ‘benchmark’ MSE values from continuous data. *Precision* is defined as the standard deviation (*SD*) of the distribution of MSE values around their mean (e.g., across simulation runs, or across subjects). (**B**) Example for an application. Extraction of respective discontinuous data segments from a block design with two conditions (**A,B**) and subsequent MSE estimation across these discontinuous segments.

In order to investigate the behavior of SampEn and MSE when reducing the number of data points and parsing continuous time series into discontinuous data segments of varying lengths (see Fig. 1), we evaluated the *accuracy* (Δ; i.e., unbiasedness) and *precision* (*SD*; i.e., dispersion) of SampEn and MSE values. Accuracy here represents the absolute deviation of (a) the average SampEn (or MSE) values of a sample of shorter or discontinuous time series from (b) the ‘benchmark’ average SampEn (or MSE) values calculated from a sample of ‘ideal’ time series with ‘optimal’ data density [i.e., Δ : = | mean(SampEn_short/discontinuous time series_) – mean(SampEn_benchmark_) |]. It allows one to approximate the bias of the SampEn and MSE values that can be expected given a certain amount of data points (continuous as well as discontinuous). Precision here is defined as the standard deviation (*SD*) of the distribution of the SampEn (or MSE) values around their mean and is equivalent to the dispersion of the accuracy at any level of data density and data segmentation. Consequently, the precision allows one to estimate the standard error of the SampEn and MSE values given a certain sample size and thus can be used to approximate the statistical power in a given experimental design^22^, as will be discussed in detail below.

### Simulations

All simulations and analyses were conducted within MATLAB R2012a and R2014b (The MathWorks Inc., Natick, MA). White noise was generated using the randn function. 1/f noise was generated using an implementation of simulating discrete noise^23^ by Stoyanov *et al.*^24^ (http://people.sc.fsu.edu/~jburkardt/m_src/cnoise/cnoise). As has been previously shown^4^, SampEn (and thus MSE) is highly accurate and precise for time series with more than 10,000 data points. Thus, we used SampEn and MSE estimated from time series with 2^16^ (65,536) data points as the benchmark for all comparisons. In order to obtain stable SampEn and MSE estimates, all reported point measures (i.e., mean (*M*), and standard deviation (*SD*)) are calculated from 1,000 randomly generated time series. Furthermore, in order to keep the spectral power at the lower end of the frequency spectrum constant for different signal lengths, time series were high-pass filtered with a frequency cutoff of *sampling frequency*/2^10^ (1,024) Hz (e.g., 1 Hz cutoff with a sampling frequency of 1,024 Hz) with a 4^th^ order Butterworth filter implemented using the filtfilt function in MATLAB.

In order to evaluate the robustness of SampEn to reductions in the number of data points, we compared the average deviation of SampEn calculated from 2^5^ (32) to 2^15^ (32,768) successive data points from the benchmark SampEn calculated from 2^16^ (65,536) successive data points. As introduced above, we refer to the absolute average deviation as *accuracy* and to the *SD* of the SampEn values as *precision*, both calculated across SampEn values of 1,000 randomly generated time series (representing randomly drawn samples of white or 1/f noise). It should be noted that smaller numerical values denote higher accuracy and precision. Since the average deviation and the *SD* decrease approximately exponentially with increasing data volume, results are plotted in log-log space. After determining the number of data points needed to obtain stable SampEn estimates, we examined the impact of discontinuous data segment lengths on SampEn. In a first pass, segments of 128, 32, 16, 8, 4, and 3 data points were used to approach the absolute limits of the behavior of SampEn when using as little continuous data as possible (i.e., as mentioned above, if *m* = 2, the minimum number of data points to compute SampEn is 2 + 1 = 3). However, because of a clear monotony of results across these segment lengths (Figure S3), for simplicity we present only segments of 128, 32, and 3 in the current results. Since the critical measure within SampEn is the frequency of reoccurring patterns, the comparison between SampEn estimation from discontinuous and continuous data was based on an equivalent number of compared patterns [number of compared patterns = (*n*−*m*) × *j,* in which *j* denotes the number of segments, *m* is the *m* parameter of the SampEn (MSE) measure, and *n* represents the number of data points within segments]. Combined, these analyses were intended to establish the critical amount of data necessary to obtain relatively stable SampEn and MSE estimates from continuous and discontinuous data.

Finally, we extended the above single-scale analyses to multiple scales (up to scale 20). MSE calculated from time series with 2^16^ (65,536) successive data points served as the benchmark. We compared estimation across 10 to 640 discontinuous segments with lengths of 2^4^ (16) to 2^10^ (1,024) data points, and a maximum number of 20 × 2^10^ (20 × 1,024) data points, to the benchmark. Accuracy and precision of the MSE estimation across discontinuous segments are again reported as the log_10_ of the absolute average difference from the benchmark MSE values, and log_10_ of the *SD* of the MSE values, respectively.

### Application to EEG Data

In order to test whether simulated results were applicable to typical neurophysiological data, we conducted similar analyses using resting state EEG data from the large-scale COGITO study conducted at the Max Planck Institute for Human Development (MPIB), Berlin, Germany (see Schmiedek *et al.*^25^ for a detailed description of the study). The study was approved by the ethics committee of the MPIB, and was carried out in accordance with the approved guidelines. All participants provided written informed consent. A detailed description of the resting state EEG data is provided in Grandy *et al.*^26^. In short, resting state EEG data were initially recorded from 45 younger adults (24 women; age range = 20–31 years; *M* = 25.1; *SD* = 2.8) with BrainAmp amplifiers (Brain Products GmbH, Gilching, Germany) and 64 Ag/AgCl electrodes. Sixty scalp electrodes embedded in an elastic cap (EASYCAP GmbH, Herrsching, Germany) were organized according to the 10% system. The EEG was recorded with an analog band-pass of 0.1 to 250 Hz and digitized with a sampling rate of 1 kHz. EEG resting state data were acquired from two conditions: 2 min eyes closed (EC) and 2 min eyes open (EO). Participants were instructed to sit as relaxed and as still as possible in both the EC and EO conditions, and to fixate on a cross during the EO condition. During recording, the ground was placed at AFz. Two electrodes were placed on the outer canthi and one electrode below the left eye in order to monitor eye movements. During recording, all electrodes were referenced to the right mastoid electrode. Electrode impedances were maintained below 5 kΩ before recording.

Preprocessing and analysis of EEG data was performed using the EEGLAB^27^ and FieldTrip^28^ toolboxes, as well as custom MATLAB code. EEG data was re-referenced to mathematically linked mastoids, high-pass filtered with a 4^th^ order Butterworth filter with a cutoff frequency of 250/2^10^ (250/1,024) Hz ( = 0.24 Hz), down-sampled to 250 Hz and segmented into epochs of 4.096 sec (i.e., 1,024 data points). Epochs were visually inspected for each subject, and all segments containing artifacts other than eye blinks and eye movements were excluded from further analyses. After manual artifact rejection, a temporal independent component analysis^29^ (ICA) was conducted. Independent components capturing eye blinks and movements were visually identified and removed prior to back-projection of the data. Only participants with at least 20 artifact free segments of 1,024 data points (4.096 sec) within each condition were kept for the current analyses, leaving *n* = 19 individuals. For each of the 19 participants, the first 20 artifact free segments were chosen for all subsequent analyses, ensuring an identical amount of data for all included individuals. Importantly, our selected subsample (*n* = 19) did not differ from excluded sample participants in terms of several relevant sample descriptives [age (*t*(43) = 0.62, *p* = 0.537), perceptual speed (Digit Symbol Substitution scores; *t*(43) = 0.39, *p* = 0.700), fluid intelligence (Raven Advanced Progressive Matrices scores; *t*(43) = 1.22, *p* = 0.231), and individual alpha frequency (*t*(43) = −1.22, p = 0.230; cf. Grandy *et al.*^26^].

The rationale for these choices was two-fold. First, we aimed at obtaining a sample size within the range of a typical cognitive neuroscience experiment (*N* ≈ 20). Second, we sought a number of available data points that would allow highly accurate MSE estimates, to serve as a valid benchmark for assessing the effects of reducing the number of data points in a neurophysiological signal. It was not possible to obtain 10,000 successive data points in our EEG data, although in our simulations the bias of MSE estimates from 10,000 successive data points was below 0.001 up to scale 20 when estimating the MSE across 20 segments of 1,024 data points (see Results). As a result, we used 20 × 2^10^ (20 × 1,024) data points (instead of time series with successive data) as the benchmark for EEG data in the current study. The amount of data was then systematically reduced from 1/2 to 1/2^7^ (1/128) by sampling between *j* discrete segments (*j* = 10 to 640) with lengths of *n* data points [*n* = 2^4^ (16) to 2^10^ (1,024)] from the overall pool of 20 × 2^10^ (20 × 1,024) available data points. MSE was calculated for *j* × *n* data points for every electrode. For accuracy, the log_10_ of the absolute average deviation from the benchmark MSE, averaged across all electrodes and participants (representing a random sample of resting-state EEG signals), is reported. For precision, the average *SD* calculated across participants’ MSE values within electrodes is reported. To provide the lower limits of accuracy and precision, the 95^th^ percentiles and maximum values of the respective distributions are also presented.

Furthermore, the robustness of the statistical difference between the two resting conditions (EC vs. EO) when reducing the amount of data used for MSE estimation was investigated to demonstrate more practical implications for typical cognitive neuroscience study designs. Point-wise (scales × electrodes) paired-samples Student’s *t*-tests (EC vs. EO) were conducted for all *j* × *n* data sets. We then calculated the *sensitivity* and *specificity* of detecting a reliable difference between conditions (with the *α*-level set at 0.05) as a function of decreasing the overall amount of data. In the current context, sensitivity denotes the probability to detect a reliable difference between conditions (across electrodes and scales) given that the benchmark comparison of the two conditions indicated a reliable difference. Specificity denotes the probability to obtain a non-significant difference between conditions given that the benchmark comparison rendered a non-significant difference. Note that the meaning of ‘sensitivity’ and ‘specificity’ in our context thus deviates slightly from their use in standard diagnostics; nevertheless, as introduced here, they provide a useful heuristic to describe the effect of reducing the number of data points on the statistical comparison of the two conditions.

### Application to fMRI Data

Finally, we also applied the above analyses to high-speed, multiband fMRI resting state data from 20 randomly selected young adults (age range = 20–30 years) from the NKI-Enhanced dataset (publicly available at http://fcon_1000.projects.nitrc.org/indi/enhanced/download.html). All participants were right-handed and reported to be psychiatrically and neurologically healthy. As noted in Nooner *et al.*^30^, Institutional Review Board Approval was obtained for the NKI-Enhanced project at the Nathan Kline Institute (Phase I #226781 and Phase II #239708) and at Montclair State University (Phase I #000983A and Phase II #000983B), and the study was carried out in accordance with the approved guidelines. Written informed consent was obtained for all study participants.

Whole-brain resting-state fMRI data (10 mins, 900 volumes total) were collected via a 3T Siemens TrioTim MRI system (Erlangen, Germany) using a multi-band EPI sequence (TR = 645 ms; TE = 30 ms; flip angle 60°; FoV = 222 mm; voxel size 3 ×3 ×3 mm; 40 transverse slices; for full scanning protocol, see http://fcon_1000.projects.nitrc.org/indi/pro/eNKI_RS_TRT/ Rest_645.pdf). The first 15 volumes (15 × 645 ms = 9.7 sec) were removed to ensure a steady state of tissue magnetization (total remaining volumes = 885). A T1-weighted structural scan was also acquired to enable co-registration to functional images (MPRAGE: TR = 1900 ms; TE = 2.52 ms; flip angle 9°; FoV = 250 mm; voxel size 1 × 1 × 1 mm; 176 sagittal slices; full details at http://fcon_1000.projects.nitrc.org/indi/enhanced/NKI_MPRAGE.pdf).

fMRI data were preprocessed with FSL 5^31^,^32^. Pre-processing included motion-correction with spatial smoothing (7 mm full-width at half maximum Gaussian kernel) and high-pass filtering (sigma = 100 sec). We registered functional images to participant-specific T1 images, and from T1 to 2mm standard space (MNI 152_T1) using FLIRT. We then masked the functional data with the GM tissue prior provided in FSL (thresholded at probability >0.37). We detrended the data (up to a cubic trend) using the “SPM_detrend” function in SPM8. We also utilized extended preprocessing steps to further reduce data artifacts^33^,^34^,^35^,^36^. Specifically, we subsequently examined all functional volumes for artifacts via independent component analysis (ICA) within-person, as implemented in FSL/MELODIC^37^. Noise components were targeted according to several key criteria: a) Spiking (components dominated by abrupt time series spikes ≥6 *SD*s); b) Motion (prominent edge or “ringing” effects, sometimes [but not always] accompanied by large time series spikes); c) Susceptibility and flow artifacts (prominent air-tissue boundary or sinus activation; typically represents cardio/respiratory effects); d) White matter (WM) and ventricle activation; e) Low-frequency signal drift; f) High power in high-frequency ranges unlikely to represent neural activity (≥75% of total spectral power present above 0.13 Hz;); and g) Spatial distribution (“spotty” or “speckled” spatial pattern that appears scattered randomly across ≥25% of the brain, with few if any clusters with ≥10 contiguous voxels [at 4 × 4 × 4 mm voxel size]). By default, we utilize a conservative set of rejection criteria; if manual classification decisions were difficult due to the co-occurrence of apparent “signal” and “noise” in a single component, we typically elected to keep such components. Three independent raters of noise components were utilized; >90% inter-rater reliability was required on separate data before denoising decisions were made on the current data. Components identified as artifacts were then regressed from corresponding fMRI runs using the FSL regfilt command.

As 885 volumes were available for each voxel, this in principle provided sufficient data for the stable estimation of the MSE for approximately scales 1 through 10 (cf. Pincus and Goldberger^15^). MSE values calculated from 885 volumes served as the benchmark. We then tested the behavior of the MSE estimate when computing MSE across *j* ( = 10, 20, 40) discontinuous segments of *n* ( = 16, 32, 64) data points, with a maximum number of 10 × 64 data points. For accuracy (and comparable to our EEG data analyses), the log_10_ of the absolute average deviation from the benchmark MSE, averaged across all voxels and participants (representing a random sample of resting state fMRI signals), is reported. For precision, the log_10_ of the average *SD* calculated across participants’ MSE values within voxels is reported. To provide the lower limits of accuracy and precision the 95^th^ percentiles and maximum values of the respective distributions across voxels are also shown.

Furthermore, we investigated if there was any evidence for a systematic difference (or bias) in the accuracy and precision across voxels. To do so, we calculated for each *j* × *n* combination and scale whether the voxel-wise accuracy deviated reliably (uncorrected *p* = 0.001) from the overall accuracy (average across participants and voxels). As *t* values are defined as *t*(*df*) : = (voxel-wise accuracy−overall accuracy)/(voxel-wise precision/*N*^1/2^), with *df* = 19 and *N* = 20, this rationale effectively represents an integration of the accuracy and precision metrics across voxels.

## Results

### Simulated data

**Figure 2:**
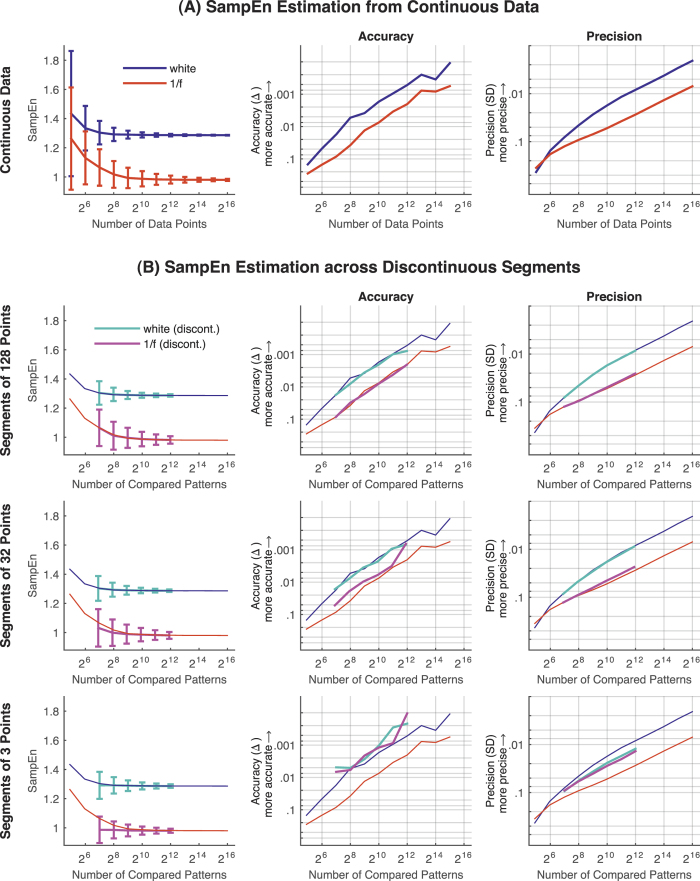
SampEn simulations using white and 1/f noise. (**A**) Accuracy and precision of SampEn (MSE at scale 1) estimates for white noise and 1/f noise as a function of signal length (number of successive data points). The average SampEn across 1,000 randomly generated time series with 2^16^ (65,536) continuous data points served as the benchmark for accuracy estimation. (**B**) Accuracy and precision of SampEn estimates for white noise and 1/f noise as a function of number of pattern comparisons when estimating SampEn across discontinuous segments of 128, 32, and 3 data points. For better comparability, SampEn estimates from successive data points (with equating the number of pattern comparisons, see Methods) are also provided [color coding as in (**A**)]. *Note.* Error bars indicate the *SD* of the SampEn values across 1,000 randomly generated time series, hence represent the precision of the SampEn values. Accuracy and precision are given in log_10_ scaling (with smaller values plotted upwards) for better readability. SampEn = sample entropy; SD = standard deviation; Δ = absolute average difference between benchmark SampEn and SampEn estimated from shorter or discontinuous time series; discont. = discontinuous segments.

First, we examined the accuracy (Δ; absolute average deviation) and precision (*SD*) of SampEn (i.e., MSE at scale 1) of white and 1/f noise as a function of signal length (Fig. 2A). Accuracy of the SampEn was lower—showing a systematic overestimation—for shorter time series. In log-log space, the accuracy and precision increased approximately linearly (monotonically) with data volume. Furthermore, accuracy and precision were lower for 1/f noise. Thus, importantly, the precision of the SampEn estimate not only depended on the signal length but also on signal structure.

Second, because SampEn operates on discrete data, we hypothesized that the critical quantity for an unbiased and accurate estimate of SampEn is the number of compared patterns rather than the number of continuous data points. Convergent with this hypothesis, accuracy and precision of SampEn were comparable for continuous and discontinuous time series when holding the number of compared patterns constant (Fig. 2B). Even under a minimal segment length of only three successive data points, accuracy of the SampEn estimate was exceptionally high when estimating across a sufficient number of segments. Interestingly, the accuracy became consistently higher when estimating the SampEn across shorter segments, relative to the SampEn estimated from continuous data with an equal amount of pattern comparisons, and this phenomenon was more pronounced for 1/f noise. In a similar vein, for 1/f noise, precision of SampEn was higher when estimated across segments (see also Figure S3).

**Figure 3:**
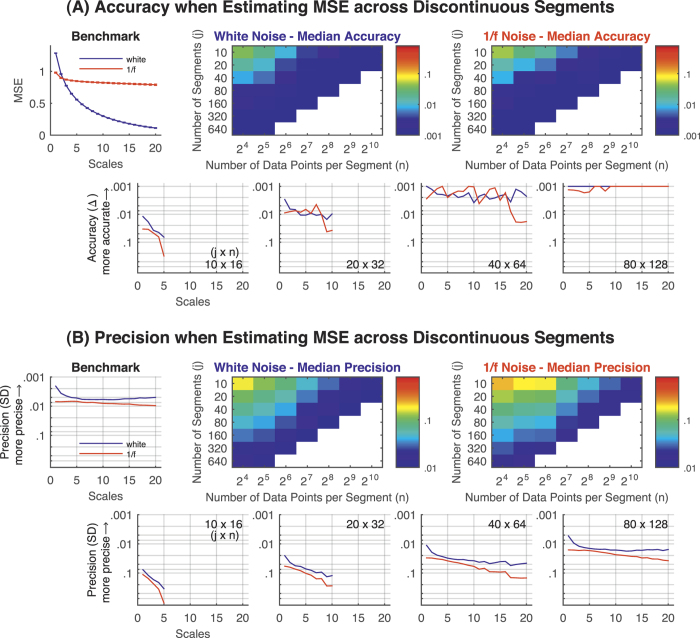
MSE simulations using white and 1/f noise. Accuracy and precision of MSE estimates for white and 1/f noise up to scale 20 when estimating the MSE across *j* ( = 10 to 640) discontinuous segments of *n* ( = 16 to 1,024) data points. (**A**) Accuracy. The average MSE values across 1,000 randomly generated time series with 2^16^ (65,536) continuous data points served as the benchmark for accuracy estimation. Benchmark MSE values are shown in the upper left plot. Heat maps provide the median accuracy across scales for each combination of *j* segments of *n* data points; MSE values were estimated only for non-white cells. Color bars indicate the (logarithmic) scaling of the median accuracy values. Note that diagonals from the bottom left to the top right refer to approximately the same number of pattern comparisons. In the lower row, representative examples of *j* × *n* combinations are provided. (**B**) Precision. Heat maps provide the median precision across scales for each combination of *j* segments of *n* data; MSE was estimated for non-white cells. Color bars indicate the (logarithmic) scaling of the median precision values. In the lower row, representative examples of *j* × *n* combinations are provided. *Note.* Error bars indicate the *SD* of the MSE values across 1,000 randomly generated time series. Accuracy and precision are given in log_10_ scaling with smaller values plotted upwards. MSE = multi-scale entropy; Δ = absolute average difference between benchmark MSE and MSE estimated across discontinuous time series with a reduced overall amount of data points; SD = standard deviation.

Next, we assessed the accuracy and precision of the MSE (i.e., “SampEn” estimated at multiple scales) when estimated across discontinuous segments. The overall observed pattern was well in line with our simulated SampEn results above. Accuracy and precision systematically increased with the number of data points and pattern comparisons available for MSE (Fig. 3, S4, and S5). Furthermore, accuracy and precision of MSE values were higher for white noise, particularly for higher scales. Thus, MSE estimates across multiple scales are also comparable for discontinuous and continuous data when ensuring a sufficient amount of data. Of note, an important consequence of our approach is that it allows the estimation of scales not accessible from the native segment length. For example, calculation of scale 20 would not be possible with only 128 data points, but can be estimated with exceptional accuracy when estimating across multiple segments of 128 data points for white and 1/f noise (Fig. 3 and S4).

### Resting-state EEG and fMRI data

**Figure 4:**
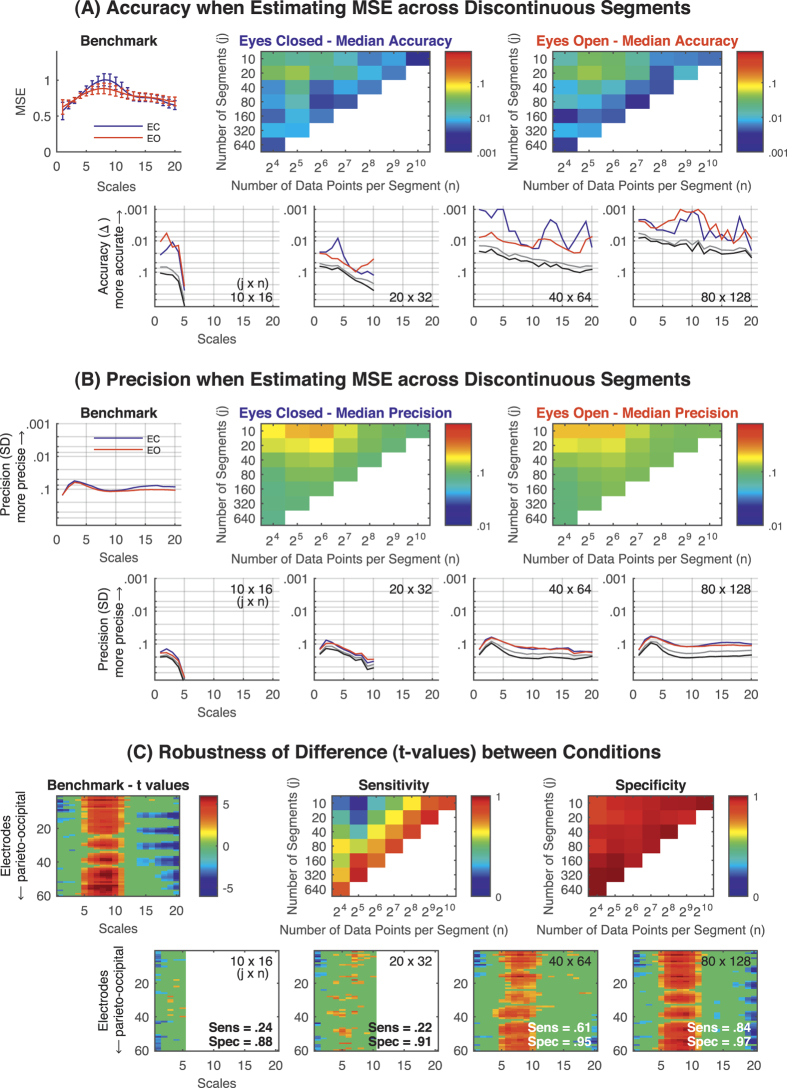
Resting state EEG data [eyes closed (EC) and eyes open (EO)]. (**A**) Accuracy and (B) Precision. MSE estimates—up to scale 20—across *j* ( = 10 to 640) discontinuous segments of *n* ( = 16 to 1,024) data points. MSE values estimated across 20 × 2^10^ (20 × 1,024) data points, averaged across all electrodes and participants (*N* = 19) served as the benchmark [shown in the upper left plots of (**A**) and (**B**)]. Heat maps provide the median accuracy (**A**) and precision (**B**) across scales for each *j* × *n* combination. Color bars indicate the (logarithmic) scaling of values. Diagonals from the bottom left to the top right refer to approximately the same number of pattern comparisons. In the lower row, representative examples of *j* × *n* combinations are provided. Grey lines indicate the 95^th^ percentile and black lines the maximum (worst) accuracy and precision values across electrodes, respectively. (**C**) Robustness of statistical differences between conditions. Upper left heat map: *t* values from the MSE comparison between EC and EO. Non-significant scale × electrode points are masked out (green). Upper middle and right heat map: sensitivity and specificity of detecting significant differences between conditions across the scale × electrode space when reducing the amount of data used for MSE estimation. Sensitivity systematically decreases with decreasing amounts of data without introducing many false positives (stable specificity). The lower row provides representative *t* value maps for four *j* × *n* combinations; same scaling as the benchmark *t* value map. Note, that for short segments (i.e., 16 data points), scales containing the maximum difference between conditions cannot be obtained (e.g., lower row, leftmost plot). *Note.* Error bars indicate the *SD* of the average MSE values across participants (*N* = 19). Accuracy and precision are given in log_10_ scaling with smaller values plotted upwards. In heat maps, values were estimated only for non-white cells. MSE = multi-scale entropy; Δ = absolute average difference between benchmark MSE and MSE estimated across discontinuous time series with a reduced overall amount of data points; SD = standard deviation; Sens = sensitivity; Spec = specificity.

Following our simulations, we evaluated whether the above observations could be extended to EEG data. MSE estimated from 20 × 2^10^ (20 × 1,024) data points served as the benchmark for the resting state EEG data. Figure 4 provides the accuracy and precision of MSE calculated from less than 20 × 2^10^ (20 × 1,024) data points, averaged across 60 electrodes. Overall, accuracy and precision were comparable to the simulated data (see also Figures S6 and S7) and approached benchmark levels. Again, even when estimating across segments with limited numbers of data points, accuracy and precision of the MSE estimates were high with a sufficient number of segments. Thus, our simulated results can be extended to realistic neurophysiological time-series. In a final step, we tested how well MSE differences between the EC and EO conditions could be detected when using increasingly fewer and shorter discontinuous segments (Fig. 4C). Unsurprisingly, the statistical difference between the two resting conditions was found to be increasingly robust with more data. For the equivalent of 10 × 2^10^ (10 × 1,024) data points, the sensitivity to reliably detect an existing difference between conditions ranged between 0.77 and 0.94, for 10 × 2^9^ (10 × 512) data points between 0.68 and 0.90, for 10 × 2^8^ (10 × 256) data points between 0.57 and 0.65, and for 10 × 2^7^ (10 × 128) data points between 0.37 and 0.65. Thus, the overall pattern of differences between the EC and EO condition was highly robust, but in line with the accuracy and precision reported above, robustness of the difference was increasingly limited when estimating the MSE from fewer data points (see also Figure S8). On the other hand, specificity remained high (>0.88) throughout, that is, the probability of falsely detecting reliable differences between conditions (or groups) did not substantially change when reducing the overall amount of data.

**Figure 5:**
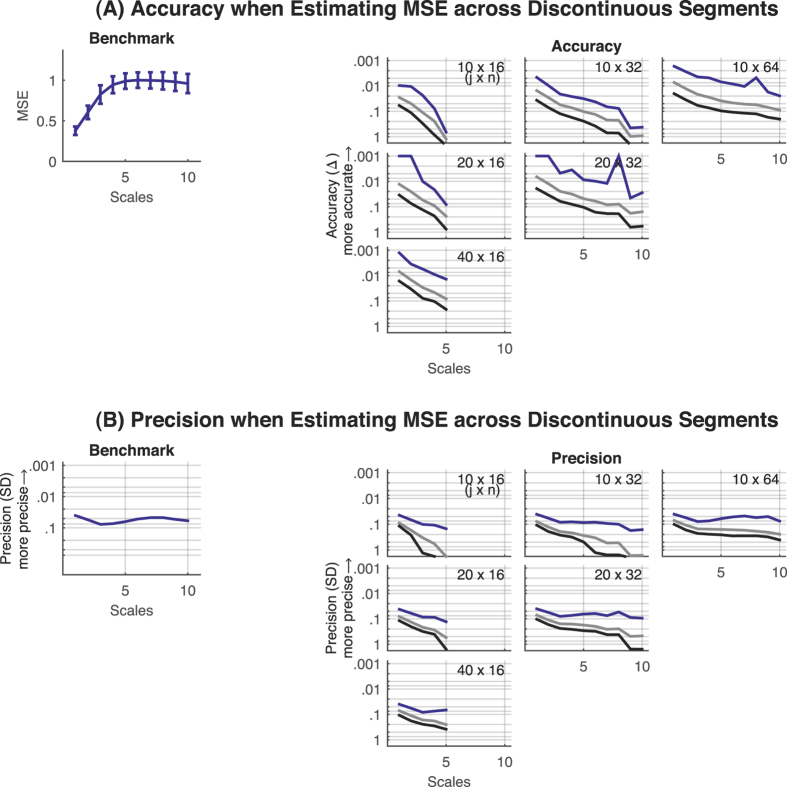
Resting state fMRI data. Accuracy and precision of the MSE estimates up to scale 10 when estimating the MSE across *j* ( = 10 to 40) discontinuous segments of *n* ( = 16 to 64) data points of resting state fMRI data. (**A**) Accuracy. MSE values estimated from 885 continuous data points and averaged across all voxels and participants (*N* = 20) served as the benchmark for accuracy estimation. Benchmark MSE values are shown in the upper left plot. All possible combinations of *j* segments of *n* data points are shown in the right panels. (**B**) Precision. Precision of the benchmark MSE values is shown in the upper left plot. *Note.* Error bars indicate the *SD* of the average MSE values across participants (*N* = 20). The grey lines indicate the 95^th^ percentile and the black lines the maximum values (worst case) of accuracy and precision values across voxels, respectively. Accuracy and precision are given in log_10_ scaling with smaller values plotted upwards. MSE = multi-scale entropy; Δ = absolute average difference between benchmark MSE and MSE estimated across discontinuous time series with a reduced overall amount of data points; SD = standard deviation.

**Figure 6:**
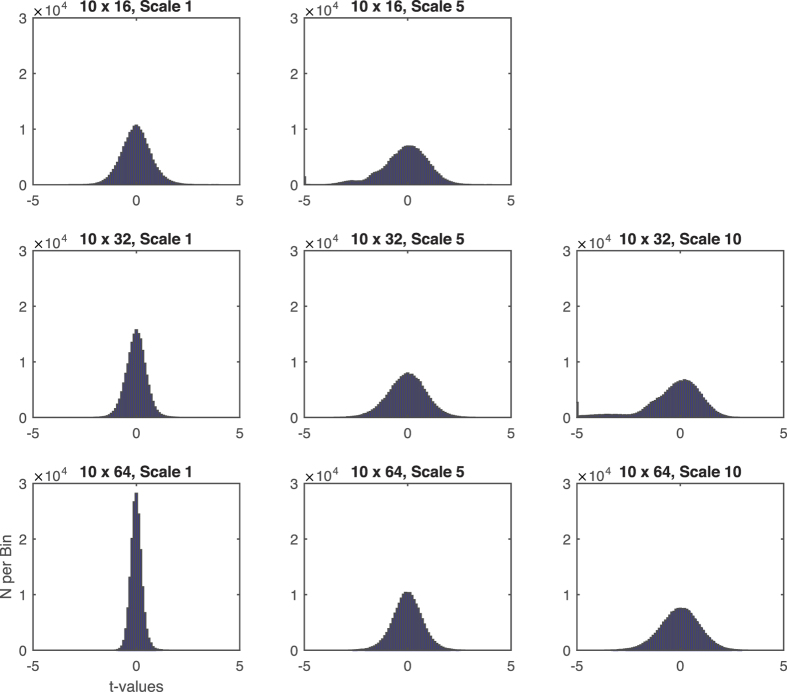
fMRI: t value distributions of comparing voxel-wise accuracy with overall accuracy (for exemplary j × n combinations and scales). Regardless of the number of data segments, data points per segment, and time scale, deviation of the voxel-wise accuracy from overall accuracy were effectively zero-centered, with tighter and more symmetric distributions at higher data densities (scale 1 vs. higher scales; lower vs. higher number of data points per segment).

**Figure 7:**
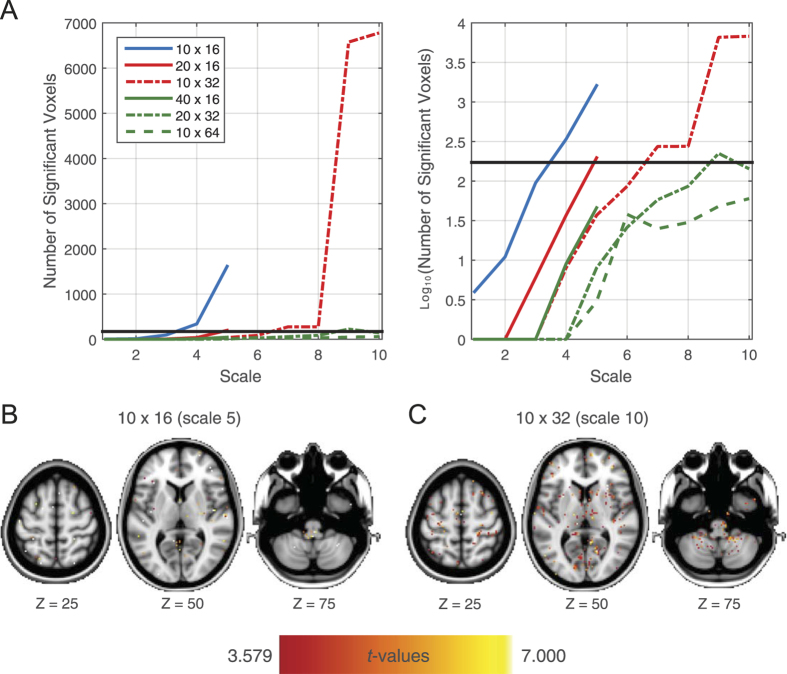
fMRI: number and spatial distribution of voxels where accuracy deviated reliably from the overall accuracy. (A) Number [and log_10_(number)] of reliably deviating voxels as a function of time scale and *j* (number of data segments) × *n* (data points per segment) combination. The black horizontal line indicates the number of voxels that would be detected by chance at an uncorrected *p* of 0.001. Note, that the blue, red, and green lines correspond to 160, 320, and 640 overall data points (across segments), respectively, thus denoting approximately the same amount of pattern comparisons. (B) and (C) The spatial distribution of the reliably deviating accuracy values from the overall accuracy (as indexed by the *t* values) for the two worst cases [(B): 10 × 16, scale 5; (C): 10 × 32, scale 10] indicates that accuracy values are randomly distributed across the brain.

Finally, we examined this phenomenon in resting-state fMRI data. Again, MSE accuracy and precision increased with greater overall amount of data, independent of the length of the discontinuous segments (Fig. 5). Importantly, the conjoint evaluation of a systematic bias of accuracy and precision of MSE values across voxels did not reveal any area where the voxel-level accuracy deviated systematically from the overall accuracy. The distribution of *t* values are symmetric around zero and follow the general trend of more narrow (i.e., more consistent) distributions with larger amounts of data (Fig. 6). When the amount of available patterns for comparisons are sufficient, the amount of voxels that reliably differ in their accuracy from the overall accuracy is below the number expected by chance (Fig. 7A). Furthermore, even when a large amount of voxels show reliable differences (worst case scenario at the absolute limits of MSE estimation, i.e. number of compared patterns << 100), the spatial distribution appears random (Fig. 7B and C).

## Discussion

In the current paper, we introduced and evaluated a solution to the data density requirements for SampEn and MSE, with the intention of yielding greater applicability of SampEn and MSE to sparse neurophysiological data (e.g., EEG data with short trials or fMRI data from block or event-related designs). Given the discrete nature of these entropy measures, we hypothesized that it would be possible to calculate SampEn and MSE across discontinuous data segments with high accuracy and precision; this hypothesis was supported across simulation-based, EEG, and fMRI analyses.

### Simulations

We started by describing and evaluating the accuracy (absolute average deviation) and precision (*SD*) of the SampEn (MSE at scale 1) as a function of signal length using simulated white and 1/f noise. Both accuracy and precision of SampEn were found to improve approximately linearly (monotonically) in log-log space as a function of signal length (Fig. 2A), emphasizing the importance of the number of data points for the robust estimation of SampEn. For increasingly shorter time series an increased overestimation in the exact SampEn value was observed and the level of overestimation was higher for 1/f noise (Fig. 2). The difference in overestimation is most likely driven by differences in frequency content between the two noise types. As white noise has a flat spectrum (and thus a more consistent time series variance), sampling fewer data points has relatively little impact on the estimation of the *SD* of the time series. However, for 1/f noise which is characterized by slower rather than faster frequency content, reducing the number of continuous data points necessarily reduces the low frequency content that is captured and hence reduces the *SD* estimate. Thus, for sparser time series from 1/f noise, the boundary interval (*r* × *SD*) over which data points are compared is smaller and, holding all else equal, has the effect of increasing the entropy value. In line with this notion, no difference in overestimation is observed when estimating SampEn with the *SD* of the overall time series from which the shorter segment was sampled (see Figure S9).

For real data, potential properties and differences in SampEn and MSE values between signals are likely unknown but may be rather subtle, ensuring that any given signal(s) will fall somewhere along the log-log continuums we report for accuracy and precision. Thus, in any given study design, such overestimation is not necessarily problematic *per se*, but rather underscores the necessity of ensuring equal numbers of data points across conditions and subjects in comparative settings so that any overestimation bias remains stable. Hence, we cannot recommend a fixed number of data points as a required minimum for accurate and precise estimation of SampEn and MSE; similar to how choices of sample size impact statistical power, the number of required data points strongly depends on the requirements of a given study’s analyses. We provide a more detailed discussion of various practical implications below.

We then assessed the effect of calculating SampEn and MSE across discontinuous segments. As hypothesized, when equating the number of pattern comparisons, estimates across discontinuous segments were as accurate and precise as those from continuous time series. Importantly, even with a segment length at the absolute minimum (of *n* = *m* + 1 = 3 in the current report), accuracy was exceptionally high when including a sufficient number of segments. Thus, neither discontinuity nor segment length impeded accurate estimation of SampEn or MSE.

### Validation in EEG and fMRI data

Next, we evaluated these issues in relation to real neurophysiological data (EEG and fMRI). The overall picture for the EEG data was comparable to our simulation-based results. Accuracy and precision of MSE estimates were higher, albeit lower than in the simulations, with increasing number of data points and pattern comparisons (Fig. 4A,B). Since the accuracy of the MSE estimate per se is not necessarily meaningful as an explicit value, we also heuristically assessed the practical consequences of the inaccuracies when limiting the amount of data points when comparing EO and EC conditions. Robustness of the statistical difference was assessed by calculating the sensitivity and specificity (as defined in the Methods section) of detecting reliable differences between conditions. Sensitivity was high (between 0.77 and 0.94) when using only half the data points [equivalents of 10 × 2^10^ (10 × 1,204) data points], but decreased when further reducing the amount of data included in the MSE estimation. Notably though, sensitivity was not dependent on the length of the discontinuous segments (Fig. 4C). On the other hand, specificity remained high despite reducing the overall amount of data. Thus, decreasing the amount of data decreases the probability of detecting reliable differences between conditions (decreased sensitivity) without introducing increasingly false positives (stable specificity). That is, while the probability of (falsely) detecting reliable differences between conditions (or groups) does not change, the probability of detecting existing ‘true’ differences is greatly reduced when reducing the amount of data.

Finally, we extended the applicability of our approach to fMRI data, for which acquisition of continuous data is typically sparse due to an impoverished sampling rate [albeit here, 885 multiband volumes were collected within 10 minutes (TR = 645 ms), serving as an example that approaches current best-case scenarios for temporal resolution of the BOLD signal while maintaining adequate spatial resolution]. As expected, high MSE accuracy and precision were achieved when including sufficient data in the MSE estimation. Furthermore, no evidence was found for a systematic influence of voxel location on accuracy and precision. The larger the number of data points available for MSE estimation, the less the number of voxels with reliable deviations from the overall accuracy. Importantly, even in the worst-case scenarios, accuracy values appeared to be randomly distributed across voxels (Fig. 7). This indicates an ideal scenario in that the reported mean accuracies do provide a representative approximation of the accuracy across the whole brain. As our reported accuracy and precision values refer to a voxel size of 2 × 2 × 2 mm, accuracy and precision values would likely improve even further if anatomical or functional parcellation schemes were used (such as AAL). In this sense the voxel-level values may be conceived of as an effective lower bound on regional estimates.

### Benefits of discontinuous MSE estimation

Of note, an interesting and important consequence of our general approach is that it allows the estimation of scales not accessible from native data segment lengths. For example, calculation of scale 20 is not possible from only 128 data points, but was estimated highly accurately in our simulations when estimating the MSE across multiple segments of 128 data points. This is an important advance for some classes of neurophysiological data. For example, in many typical EEG (e.g. short trials, or short time series due to artifacts) or fMRI settings (TR = 2 sec), the number of successive data points is severely limited. However, in contrary to some signal types (e.g., heart rate data), scale 1 (i.e., the sampling frequency) is often not the most interesting scale in M/EEG or fMRI data. With a limited number of successive data points, the scales of interest that map, for example, to the alpha (8–12 Hz) or theta frequencies (4–7 Hz) may not normally be accessible. Thus, our approach of MSE estimation across discontinuous segments allows inclusion of higher scales that were previously not estimable, permitting one to map out an even broader range of neurophysiological dynamics.

### Why does MSE estimation from discontinuous segments work?

It may at first glance seem counterintuitive that sampling shorter data segments (or even reordering them) could yield similar MSE results as in continuous data. However, our proposed MSE solution works explicitly because data patterns are *not* computed across data segment boundaries. In this way, data patterns within each segment are “protected” and in their original state. When discrete data patterns are counted in MSE, the algorithm does not care for when in the time series data patterns occurred, but rather only that the patterns exist; because we ensure that all data patterns are real (i.e., from within data segments) rather than artificial (i.e., those crossing data segment boundaries), accurate and precise MSE estimation is indeed possible. However, “real” patterns here are necessarily bound by minimal data point requirements within segment to enable MSE estimation (*m* + 1 = 3, with or without coarse-graining); this means that only those time scales that meet this data minimum can be estimated, with the provision that enough pattern exemplars (and thus, data segments) are available in the data.

### Practical recommendations derived from the current study

Importantly, from an application point of view, our definitions of accuracy and precision allow one to approximate the sample size and/or number of data points required to detect an expected difference in MSE with a given level of statistical power^22^. We will briefly outline the basic rationale here for an example two-group comparison design (e.g., comparing healthy individuals and patients) in order to demonstrate how to make use of reported accuracy and precision values from the current study. With respect to accuracy, there are two important considerations. First, as already mentioned above, the systematic bias (i.e., the overestimation of MSE values; e.g., Fig. 2A leftmost plot) for shorter time series is approximately equal for signals with similar signal characteristics (see also Fig. 4 and S6). Consequently, as long as signal characteristics are not fundamentally different (e.g., white versus 1/f noise), the bias in the absolute MSE values should be comparable across groups (i.e., for both groups, the absolute MSE value is shifted by approximately the same amount), and should not greatly influence the statistical comparison (as MSE values in both groups are shifted in the same direction by approximately the same amount). If, however, signals do differ fundamentally in their signal characteristics, it can be expected (or should be ensured by means of including a sufficient number of data points) that the difference between the MSE values of the two signals is large compared to the difference in accuracy (see Fig. 2A: the difference between white and 1/f noise is larger than 0.3; yet, their respective accuracies are smaller (i.e., better) than 0.1). In sum, accuracy should only in rare instances impede statistical comparisons between groups. Second, and more importantly, the accuracy does show a strong dependency on the number of data points; shorter time series exhibit systematically larger MSE values. Consequently, MSE values should only be compared across groups if they are calculated on the same amount of data points as otherwise artificial differences (i.e., a differential bias) between MSE values may be introduced.

Precision of the MSE values as a function of the number of data points can be used to approximate the sample sizes required to detect an MSE difference at the electrode or voxel level with a certain probability (i.e., statistical power, 1−*β*). Reported raw MSE group differences so far range approximately between 0.02 and 0.20 in the EEG literature^9^,^38^ and approximately between 0.02 and 0.15 in the fMRI literature^7^,^10^,^39^. Using our precision values to estimate effect sizes for expected group differences in MSE values (demonstrated here for expected MSE differences—derived from the literature—of 0.05, 0.10, and 0.15) allows one to approximate the required sample sizes per group to detect a group difference with a given statistical power of, for instance, 0.80, 0.90, or 0.95 (Table 1). Most importantly, apart from the actual estimated sample sizes, it can be seen that when applying MSE at the absolute minimum of the required amount of data points (as is the case here for scale 5), doubling the amount of data can reduce the required sample size up to approximately one fifth. Thus an important practical consequence of our results is that at the limits of data sparsity the number of data points can be highly efficiently traded against the sample size.

### Caveats for MSE application

As a cautionary note for future work, in the present study we closely followed the convention regarding the use of the *SD* as the criterion for the discretization of the data as introduced^3^,^4^ and practiced in the literature^6^,^7^,^8^,^9^. That is, for every set of data points, the defined proportion *r* of the standard deviation of the original time series is used as the criterion. However, the application of the MSE to neurophysiological data in this way is not entirely unproblematic and has been subject to criticism^40^. The power spectra of many *in vivo* neurophysiological signals follows a 1/f-like characteristic^13^, but large individual differences remain in exact slope of the power spectrum, and thus in the balance of low versus high frequency content of the signal^41^. Importantly, signal variance is often strongly determined by lower frequencies (which may vary widely across individuals), whereas the initial scales of the MSE reflect higher frequencies (e.g., scale 1 reflecting the sampling frequency), and progress toward the lower parts of the frequency spectrum via coarse-graining. This property of the MSE can become problematic when the spectral power of low frequencies outside the spectrum covered by the MSE systematically differs between subjects (see also above; Figure S9). Under these circumstances, differences in the MSE may be largely determined by the power of lower frequencies. Consequently, MSE should be used with caution in comparative settings where the sole focus lies on the comparison of absolute differences in MSE across conditions or groups. Future research should be conducted to establish more comprehensive solutions to this important and non-trivial caveat.

## Conclusion

Using simulated, EEG, and fMRI data, we established a clear relationship between the accuracy and precision of MSE estimates and the number of data points (pattern comparisons) utilized in the calculation of the MSE. As hypothesized, calculation of the MSE across discontinuous segments was possible with high accuracy and precision when ensuring a sufficient amount of pattern comparisons. The use cases employed in the present study (e.g., 1/f vs. white noise in simulation; eyes-open vs. eyes-closed in EEG) are meant as simple exemplars for signal- and condition-based analyses that could be conducted by scientists; however, the overarching principle here is that our approach can indeed recover whatever underlying entropy dynamics may exist in time series, despite data segment discontinuity. Given the conceptual similarity across many typical entropy measures, it is also reasonable to assume that the current SampEn-based results may apply to related measures such as approximate entropy^5^,^42^,^43^ or even rank-order-based entropy measures (e.g., permutation entropy^44^ or rank-vector entropy^45^). Finally, an important advance of our approach for neurophysiological data is that it allows the calculation of scales previously not accessible within the native segment lengths; accordingly, consideration of relevant scales with respect to neurophysiological processes is highly facilitated and may yield a broader range of applications of SampEn and MSE when gauging moment-to-moment dynamics in sparse and/or discontinuous brain imaging data.

## Additional Information

**How to cite this article**: Grandy, T. H. *et al.* On the estimation of brain signal entropy from sparse neuroimaging data. *Sci. Rep.* **6**, 23073; doi: 10.1038/srep23073 (2016).

## Supplementary Material

Supplementary Information

## Figures and Tables

**Table 1:** Required sample sizes (*N*) per group in a group comparison design as a function of the number of fMRI volumes (influencing precision) and the expected size of the raw group difference in MSE.

		Amount of Data	Scale	MSE Precision	*Estimated group-wise N* required to achieve a specific raw group difference in MSE of:		
					0.05	0.10	0.15
**Statistical Power (1−*****β***)	**0.80**	10 × 16	1	0.09	51	13	6
			5	1.80	>1000	>1000	>1000
		10 × 32	1	0.08	41	11	5
			5	0.55	>1000	474	211
		10 × 64	1	0.07	31	8	4
			5	0.25	392	98	44
		885	1	0.06	23	6	3
			5	0.10	63	16	7
	**0.90**	10 × 16	1	0.09	68	17	8
			5	1.80	>1000	>1000	>1000
		10 × 32	1	0.08	54	14	6
			5	0.55	>1000	635	282
		10 × 64	1	0.07	42	11	5
			5	0.25	525	132	59
		885	1	0.06	31	8	4
			5	0.10	84	21	10
	**0.95**	10 × 16	1	0.09	85	22	10
			5	1.80	>1000	>1000	>1000
		10 × 32	1	0.08	67	17	8
			5	0.55	>1000	785	349
		10 × 64	1	0.07	51	13	6
			5	0.25	649	163	73
		885	1	0.06	38	10	5
			5	0.10	104	26	12

*Note*. Precision is defined as the *SD* of MSE values and can therefore directly be used to approximate the effect size of the expected effect in the statistical power calculation. Precision values shown here are the 95^th^ percentiles of the precision value distributions across voxels, thus providing a rather conservative estimation of required sample sizes.
