# Supplementary material for: On the estimation of brain signal entropy from sparse neuroimaging data
Source: Sci Rep. 2016 Mar 29;6:23073. doi: 10.1038/srep23073 (PMC4810375; doi:10.1038/srep23073)
Supplement: Supplementary Information [file srep23073-s1.doc]

**On the estimation of brain signal entropy within sparse neuroimaging data**

**– Supplemental Material –**

Thomas H. Grandy, Douglas D. Garrett, Florian Schmiedek, and Markus Werkle-Bergner

## 1. Simulation – SampEn, different *r* parameters

As described in the main text, we utilized parameter settings typically reported in the neuroimaging literature, that is *m* = 2 and *r* = .50 ^1-5^. In order to show that our main conclusions regarding concurrent estimation of SampEn across discontinuous segments are also valid for different *r* parameters, we provide results from equivalent simulations conducted with *r* = .30 (**Figure S1**) and *r* = .15 (**Figure S2**). Furthermore, for *r* = .50 we provide a more comprehensive picture for the estimation of SampEn across discontinuous segments of lengths *n* = 32, 16, 8, 4, and 3 (**Figure S3**).

## 2. Simulation – Multiscale Entropy and Discontinuous Data Segments

In the main text, we presented only a representative selection of MSE accuracy and precision levels when concurrently estimating MSE across discontinuous segments of various lengths. Here, we present a more detailed picture of accuracy (**Figure S4**) and precision (**Figure S5**) values for all evaluated combinations of *j* (= 10 to 640) segments of *n* (= 16 to 1,024) data points.

## 3. Resting State EEG Data – Multiscale Entropy and Discontinuous Data Segments

Equally, for the resting state EEG data, we present a more detailed picture of accuracy (**Figure S6**) and precision (**Figure S7**) values for all evaluated combinations of *j* (= 10 to 640) segments of *n* (= 16 to 1,024) data points. In addition, we also show the *t* value maps of the point wise (electrode × scale) comparisons between resting conditions (eyes closed vs. eyes open). The sensitivity and specificity of reliably detecting differences between conditions across the whole *t* value map as compared to the benchmark is provided in each upper right corner of the *t* value maps (**Figure S8**; upper value = sensitivity, lower value = specificity).

|  |
| --- |
| **Figure S1.** Simulations, white and 1/f noise; *r* parameter = .30. The overall pattern is well comparable to the simulations with *r* parameter = .50 (Figure 2). (A) Accuracy and precision of SampEn estimates for white noise and 1/f noise as a function of signal length (number of successive data points). The average SampEn across 1,000 randomly generated time series with 2^16^ (65,536) continuous data points served as the benchmark for accuracy estimation. (B) Accuracy and precision of SampEn estimates for white noise and 1/f noise as a function of number of pattern comparisons when concurrently estimating SampEn across *j* discontinuous segments of 128, 32, and 3 data points. For better comparability SampEn estimates from successive data points (with equating the number of pattern comparisons, see Methods) are also provided [color coding as in (A)]. *Note.* Error bars indicate the *SD* of the MSE values across 1,000 randomly generated time series, hence represent the precision of the SampEn values. Accuracy and precision are given in log_10_ scaling with smaller values plotted upwards for better readability. SampEn = sample entropy; SD = standard deviation; Δ = absolute average difference between benchmark SampEn and SampEn estimated from shorter or discontinuous time series; discont. = discontinuous segments. |
|  |
| **Figure S2.** Simulations, white and 1/f noise; *r* parameter = .15. The overall pattern is well comparable to the simulations with *r* parameter = .50 (Figure 2). (A) Accuracy and precision of SampEn estimates for white noise and 1/f noise as a function of signal length (number of successive data points). The average SampEn across 1,000 randomly generated time series with 2^16^ (65,536) continuous data points served as the benchmark for accuracy estimation. (B) Accuracy and precision of SampEn estimates for white noise and 1/f noise as a function of number of pattern comparisons when concurrently estimating SampEn across *j* discontinuous segments of 128, 32, and 3 data points. For better comparability SampEn estimates from successive data points (with equating the number of pattern comparisons, see Methods) are also provided [color coding as in (A)]. *Note.* Error bars indicate the *SD* of the MSE values across 1,000 randomly generated time series, hence represent the precision of the SampEn values. Accuracy and precision are given in log_10_ scaling with smaller values plotted upwards for better readability. SampEn = sample entropy; SD = standard deviation; Δ = absolute average difference between benchmark SampEn and SampEn estimated from shorter or discontinuous time series; discont. = discontinuous segments. |
|  |
| **Figure S3.** Simulations, white and 1/f noise; *r* parameter = .50. Accuracy and precision of SampEn estimates for white noise and 1/f noise as a function of number of pattern comparisons when concurrently estimating SampEn across *j* discontinuous segments of 32, 16, 8, 4, and 3 data points. For better comparability SampEn estimates from successive data points (with equating the number of pattern comparisons, see Methods) are also provided (white noise = blue, 1/f noise = red). *Note.* Error bars indicate the *SD* of the MSE values across 1,000 randomly generated time series, hence represent the precision of the SampEn values. Accuracy and precision are given in log_10_ scaling with smaller values plotted upwards for better readability. SampEn = sample entropy; SD = standard deviation; Δ = absolute average difference between benchmark SampEn and SampEn estimated from shorter or discontinuous time series; cont. = continuous data; discont. = discontinuous segments. |

|  |
| --- |
| **Figure S4.** Simulations, white and 1/f noise. Accuracy of multi-scale entropy (MSE) values up to scale 20 when concurrently estimating the MSE across *j* (= 10 to 640) discontinuous segments of *n* (= 16 to 1,024) data points. Average MSE values across 1,000 randomly generated time series with 2^16^ (65,536) continuous data points served as the benchmark for accuracy estimation. (A) Average absolute deviation (Δ) of MSE values concurrently estimated from *j* segments of *n* successive data points from benchmark MSE values [(B)]. Average is taken across MSE values from 1,000 randomly generated time series. Note that diagonals from the bottom left to the top right refer to approximately the same number of pattern comparisons. Deviations smaller than .001 are numerically presented as .001 for better readability. *Note.* Error bars indicate the *SD* of the MSE values across 1,000 randomly generated time series; accuracy is displayed in log_10_ scaling with smaller values plotted upwards. |
|  |
| **Figure S5.** Simulations, white and 1/f noise. Precision of multi-scale entropy (MSE) values up to scale 20 when concurrently estimating the MSE across *j* (= 10 to 640) discontinuous segments of *n* (= 16 to 1,024) data points. Precision is estimated from the *SD* of MSE values across 1,000 randomly generated time series. Benchmark precision was estimated from time series of 2^16^ (65,536) continuous data points. Note that diagonals from the bottom left to the top right refer to approximately the same number of pattern comparisons. *Note.* Precision is displayed in log_10_ scaling with smaller values plotted upwards. |
|  |
| **Figure S6.** Resting EEG data; eyes closed (EC) and eyes open (EO). Accuracy of multi-scale entropy (MSE) values up to scale 20 when concurrently estimating the MSE across *j* (= 10 to 640) discontinuous segments of *n* (= 16 to 1,024) data points. MSE values estimated from 20 × 2^10^ (1,024) data points averaged across electrodes and participants served as the benchmark for accuracy estimation (see Methods). (A) Average absolute deviation (Δ) of MSE values concurrently estimated from *j* segments of *n* successive data points from benchmark MSE values [(B)]. Average is taken across electrodes and participants. Note that diagonals from the bottom left to the top right refer to approximately the same number of pattern comparisons. Deviations smaller than .001 are numerically presented as .001 for better readability. *Note.* Error bars indicate the *SD* of the MSE values across 19 participants; accuracy is displayed in log_10_ scaling with smaller values plotted upwards. |
|  |
| **Figure S7.** Resting EEG data; eyes closed (EC) and eyes open (EO). Precision of multi-scale entropy (MSE) values up to scale 20 when concurrently estimating the MSE across *j* (= 10 to 640) discontinuous segments of *n* (= 16 to 1,024) data points. Precision is estimated from the *SD* across participants of average MSE values across electrodes. Benchmark precision was estimated from MSE values concurrently calculated from 20 × 2^10^ (1,024) data points. Note that diagonals from the bottom left to the top right refer to approximately the same number of pattern comparisons. *Note.* Precision is displayed in log_10_ scaling with smaller values plotted upwards. |
|  |
| **Figure S8.** Resting EEG data; eyes closed (EC) and eyes open (EO). Point-wise (scales × electrodes) comparison of the difference [*t*(18)] between MSE with eyes closed versus eyes open. *t* values at *p* > .05 are masked out (shown in green). The sensitivity and specificity of reliably detecting differences between conditions across the whole scales × electrodes space when reducing the amount of data as compared to the benchmark [estimated from 20 × 2^10^ (1,024) data points] is provided in each upper right corner of the *t* value maps (upper value = sensitivity, lower value = specificity). Color bar indicates the scaling of *t* values. Identical scaling for each sub-plot. Note that diagonals from the bottom left to the top right refer to approximately the same number of pattern comparisons. |

|  |
| --- |
| **Figure S9.** Underestimation of the *SD* of shorter time series underlies overestimation of SampEn for 1/f noise. (A) Normalized *SD* (i.e., *SD* of the original, full time series with 2^16^ (65,536) data points normalized to 1) as a function of segment length. In other words, the values shown here represent the mean *SD* and (standard deviation of the *SD*; error bars) calculated from shorter time series (of 2^x^ data points, x = {5 to 16}) sampled from the normalized original full time series. (B) Overestimation of SampEn of 1/f noise, as reported in the main text (Figure 2). (C) Overestimation disappears when using the overall time series *SD* (= 1) as the criterion for SampEn estimation—the accuracy values for white and 1/f noise are numerically closer in *C* as compared to *B*. |

## References

1. Heisz, J. J., Shedden, J. M. & McIntosh, A. R. Relating brain signal variability to knowledge representation. *Neuroimage* **63**, 1384-1392 (2012).

2. McDonough, I. M. & Nashiro, K. Network complexity as a measure of information processing across resting-state networks: evidence from the Human Connectome Project. *Frontiers in Human Neuroscience* **8** (2014).

3. McIntosh, A. R., Kovacevic, N. & Itier, R. J. Increased brain signal variability accompanies lower behavioral variability in development. *PLoS Comput Biol* **4**, e1000106 (2008).

4. McIntosh, A. R. *et al.* Spatiotemporal Dependency of Age-Related Changes in Brain Signal Variability. *Cereb Cortex* **24**, 1806–1817 (2014).

5. Yang, A. C. *et al.* Complexity of spontaneous BOLD activity in default mode network is correlated with cognitive function in normal male elderly: a multiscale entropy analysis. *Neurobiol Aging* **34**, 428-438 (2013).
